# Long-Term Trends in Microbiology and Outcomes of Pediatric Deep Neck Infections: An 18-Year Study

**DOI:** 10.7150/ijms.131187

**Published:** 2026-07-22

**Authors:** Fang-Ching Liu, Pei-Rung Yang, Yao-Te Tsai, Yao-Hsu Yang, Chia-Yen Liu, Geng-He Chang

**Affiliations:** 1 Department of Pediatrics, Jen - Ai Hospital, Taichung, Taiwan.; 2 Department of Traditional Chinese Medicine, Chang Gung Memorial Hospital, Chiayi, Taiwan.; 3 School of Traditional Chinese Medicine, College of Medicine, Chang Gung University, Taoyuan, Taiwan.; 4 Graduate Institute of Integrated Medicine, College of Medicine, China Medical University, Taichung, Taiwan.; 5 Department of Otolaryngology - Head and Neck Surgery, Chang Gung Memorial Hospital, Chiayi, Taiwan.; 6 Faculty of Medicine, College of Medicine, Chang Gung University, Taoyuan, Taiwan.; 7 Health Information and Epidemiology Laboratory of Chang Gung Memorial Hospital, Chiayi, Taiwan.; 8 Graduate Institute of Clinical Medical Sciences, College of Medicine, Chang Gung University, Taoyuan, Taiwan.

**Keywords:** pediatric deep neck infection, bacterial etiology, temporal trends, methicillin-resistant *Staphylococcus aureus* (MRSA), empiric antibiotic therapy

## Abstract

**Importance:**

Pediatric deep neck infection (DNI) is a potentially life-threatening condition. However, contemporary large-scale data describing microbial etiology and outcomes remain limited. Understanding long-term pathogen trends is essential for guiding empiric antibiotic therapy and improving outcomes.

**Objective:**

To investigate temporal changes in bacterial pathogens, treatment strategies, and outcomes of pediatric DNI across an 18-year period.

**Design, Setting, and Participants**: Retrospective multicenter cohort study using the Chang Gung Research Database (CGRD), a de-identified nationwide database from Taiwan's largest medical system. Hospitalized patients younger than 18 years with DNI from 2006 to 2023 were identified and divided into two 9-year periods (2006-2014 and 2015-2023).

**Main Outcomes and Measures:**

Demographics, comorbidities, treatment modalities, disease severity, and microbiological profiles were analyzed. Bacterial isolates were evaluated at genus and species levels, with methicillin-sensitive *Staphylococcus aureus* (MSSA) and methicillin-resistant *S. aureus* (MRSA) assessed separately.

**Results:**

Among 1,743 pediatric DNI cases (1,017 in 2006-2014; 726 in 2015-2023), antibiotic-only therapy increased (91.5% to 94.4%), surgical intervention decreased (8.5% to 5.7%), and no tracheostomies were performed. ICU admission declined (8.9% to 6.8%), mediastinal complications decreased (0.6% to 0%), and mortality remained 0.1%. *Staphylococcus* increased while *Streptococcus* decreased; *Veillonella* replaced *Peptostreptococcus* among anaerobes. MRSA became the predominant facultative anaerobic/aerobic pathogen, and *Veillonella parvula* emerged as the leading anaerobic species.

**Conclusions and Relevance:**

Over 18 years, pediatric DNI in Taiwan demonstrated a marked shift toward conservative, antibiotic-based management with sustained excellent outcomes. Concurrently, MRSA and anaerobes such as *Veillonella* emerged as dominant pathogens in culture-positive patients, highlighting the need to consider empiric regimens that adequately cover these evolving bacterial profiles specifically in severe, refractory, or surgically managed cases.

## 1. Introduction

Deep neck infection (DNI) is an acute, potentially life-threatening infection of the deep cervical fascial spaces 1,2. Prompt recognition and appropriate management are essential to prevent airway compromise, mediastinitis, sepsis, and other severe complications. While numerous studies have examined the clinical presentation and microbiology of adult DNI 1,2, pediatric cases remain relatively underexplored despite their unique diagnostic and management challenges. Despite being potentially life-threatening, the changing epidemiology of pediatric DNI has not been systematically evaluated in large-scale, nationwide cohorts.

Diagnosing DNI in children is difficult because symptoms are often nonspecific, physical examination may be limited by poor cooperation, and imaging interpretation can be challenging in younger patients 3,4. Obtaining purulent material for bacterial culture is also technically demanding, resulting in scarce microbiological data. Consequently, most pediatric DNI studies are single-center, involve small case numbers, rely on outdated data, and lack comprehensive microbiological analyses 3,4. To date, no large-scale investigation has systematically analyzed the bacterial etiology of pediatric DNI over an extended period or evaluated long-term pathogen trends.

Because DNI is a severe infectious emergency requiring timely empiric antibiotic therapy, understanding the current microbiological profile and its temporal changes in children is crucial. Up-to-date knowledge of the pathogens responsible for pediatric DNI can inform empiric antimicrobial selection, improve clinical outcomes, and guide public health strategies. Evaluating how treatment strategies and outcomes have evolved over time can further optimize management.

This study leveraged a large, multi-institutional database to investigate demographic characteristics, microbiological trends, treatment modalities, and outcomes of pediatric patients with DNI over an 18-year period, aiming to fill this knowledge gap with robust and contemporary data from Taiwan.

### Research in Context

• Prior investigations of pediatric deep neck infections (DNI) have been largely limited to single-center studies with small cohorts and outdated data.

• Comprehensive, multicenter analyses examining long-term temporal changes in the microbiological epidemiology of pediatric DNI have been lacking.

• Because pediatric DNI is predominantly managed with medical therapy, contemporary understanding of bacterial trends is essential to guide empiric antibiotic selection and optimize antimicrobial stewardship.

## 2. Materials and Methods

### 2.1 Data Source: Chang Gung Research Database (CGRD)

The CGRD is a de-identified medical database derived from the Chang Gung Memorial Hospital (CGMH) system, the largest healthcare network in Taiwan 5,6. It encompasses seven medical centers and regional hospitals distributed from northern to southern Taiwan, providing comprehensive inpatient and outpatient care across all major specialties. Because of its scale and diversity, the CGRD captures a substantial proportion of Taiwan's patient population and is recognized as a representative, nationwide database for studying disease patterns and healthcare outcomes 5. This study was approved by the Institutional Review Board of CGMH (Approval Number: 202301868B0C601).

### 2.2 Study Population

From January 1, 2006, to December 31, 2023, we retrieved data from the CGRD on hospitalized patients diagnosed with DNI. DNI cases were identified using specific International Classification of Diseases (ICD) codes, including cellulitis and abscess of oral soft tissues (Ludwig angina), parapharyngeal abscess, retropharyngeal abscess, and cellulitis/abscess of the neck (ICD-9-CM codes 528.3, 478.22, 478.24, 682.11 and corresponding ICD-10-CM codes after October 2015) 1,2.

We divided the cohort into two time periods: 2006-2014 and 2015-2023 to maintain balanced statistical power for comparing clinical management and microbiological trends across the study duration. Patients aged ≥18 years were excluded to obtain the pediatric cohort. The final study population comprised patients <18 years old hospitalized for DNI. Demographics, treatment modalities (antibiotic therapy, surgical drainage, tracheostomy), clinical outcomes (mediastinitis, length of stay, mortality), and microbiological profiles were compared between the two time periods to evaluate temporal changes.

### 2.3 Medical Comorbidities

Comorbidities were identified from inpatient or outpatient claims using ICD-9-CM and ICD-10-CM codes. Diagnoses were counted as comorbidities if documented at least once during an inpatient admission or three times in outpatient visits before the index DNI hospitalization. In pediatric patients, comorbidities of interest included diabetes mellitus (DM; ICD-9-CM code 250), chronic kidney disease (CKD; ICD-9-CM codes 585, 586, 403, 404) and autoimmune diseases [e.g., rheumatoid arthritis (RA), Sjögren syndrome (SS), systemic lupus erythematosus (SLE); ICD-9-CM codes 446.0, 446.2, 446.4-446.5, 446.7, 696.0-696.1, 710.0-710.4, 714.0-714.4], mapped to ICD-10-CM codes after October 2015 1,2. Previous studies show these comorbidities are associated with increased DNI risk and poorer outcomes, underscoring the need to adjust for them in analyses 7-10.

### 2.4 Bacterial Spectra

DNIs are typically polymicrobial, with common pathogens including facultative anaerobes such as *Streptococcus* spp. and *Staphylococcus* spp., aerobic bacteria like *Klebsiella* and *Pseudomonas*, and true anaerobes such as *Prevotella*, *Peptostreptococcus*, and *Veillonella* 1,2. Empiric therapy generally requires coverage of both facultative anaerobic/aerobic and anaerobic organisms.

In this study, all bacterial isolates from culture-positive pediatric DNI cases in the CGRD were categorized into two broad groups: (1) facultative anaerobes/aerobes and (2) anaerobes. We further analyzed the distributions of these pathogens at the genus and species levels and compared profiles between 2006-2014 and 2015-2023 to evaluate temporal changes.

### 2.5 Therapy Classification

Therapeutic approaches were identified from inpatient claims during the index hospitalization. Patients receiving intravenous antibiotic therapy with or without abscess needle aspiration but without formal surgical drainage were categorized as antibiotic treatment only. Those undergoing incision and drainage of the abscess or other operative procedures were classified as surgical intervention. Patients who underwent tracheostomy were separately identified to evaluate airway management.

### 2.6 Disease Severity and Prognosis Evaluation

Disease severity was evaluated using laboratory parameters at admission, including white blood cell (WBC) counts, C-reactive protein (CRP) levels, and band form percentages. We also examined ICU admission, tracheostomy, and mediastinal complications. Prognosis was assessed by analyzing in-hospital mortality during DNI treatment, with additional evaluation of whether death was accompanied by mediastinal complications and whether tracheostomy had been performed.

### 2.7 Statistical Analysis

Categorical variables (sex, comorbidities, treatment modalities, ICU admission, tracheostomy, mediastinal complications, mortality rates) were compared between time periods using Pearson's chi-square test or Fisher's exact test. Continuous variables (age, WBC counts, CRP levels) were analyzed using Student's t-test. All analyses were performed using SAS software, version 9.4 (SAS Institute, Cary, NC, USA), with p < 0.05 considered statistically significant.

Furthermore, a sensitivity analysis was performed by excluding data from the COVID-19 pandemic period (e.g., analyzing 2015-2019) to determine whether the observed long-term trends persisted independently of pandemic-related healthcare disruptions.

To identify independent predictors for surgical intervention and ICU admission, multivariable logistic regression models were constructed. Relevant covariates including age, gender, time period (2006-2014 vs. 2015-2023), and initial laboratory values (WBC and CRP) were included in the models. The results were expressed as adjusted odds ratios (aOR) with 95% confidence intervals (CIs).

## 3. Results

### 3.1 Study Population and Comorbidity Analysis

**Figure 1:**
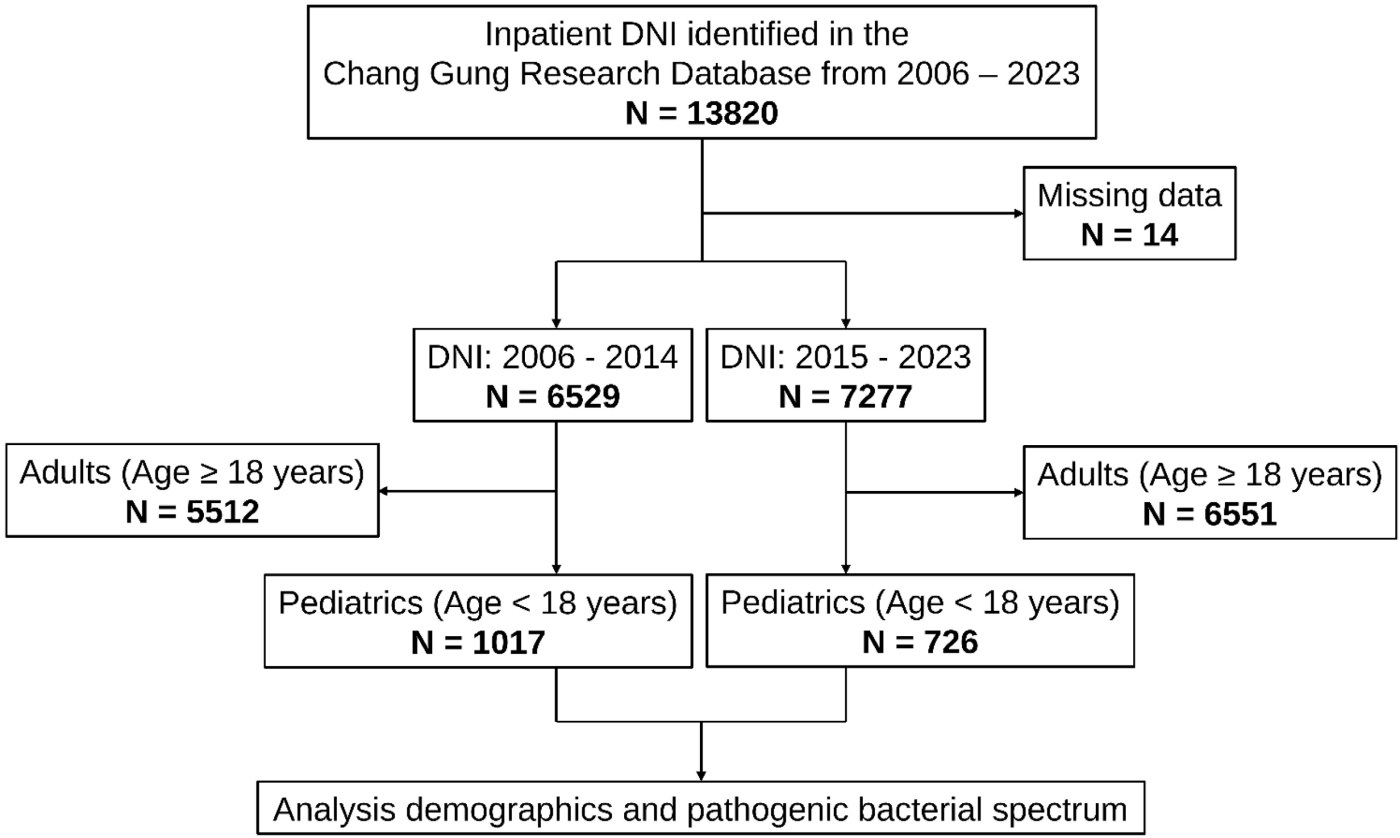
** Flowchart of Patient Selection for Pediatric DNI.** Flow diagram illustrating the selection of pediatric patients with deep neck infection (DNI) identified from the Chang Gung Research Database between 2006 and 2023.

A total of 1,743 pediatric patients (<18 years old) hospitalized for DNI were identified between 2006 and 2023 (Figure 1). After applying inclusion/exclusion criteria, 1,017 cases were included during 2006-2014 and 726 during 2015-2023.

Mean age was 7.1 ± 4.8 years in 2006-2014 and 6.7 ± 4.4 years in 2015-2023. Boys predominated in both periods (56.3% vs. 55.7%). CKD and autoimmune diseases (RA, SS, SLE) were rare. DM increased slightly in the later period (0.8% vs. 0.1%, p=0.023). No significant differences were observed in other comorbidities (Table 1).

### 3.2 Treatment Outcomes and Disease Severity

During 2006-2014, 91.5% of patients received antibiotic therapy with or without abscess aspiration, and 8.5% underwent surgical intervention. In 2015-2023, antibiotic therapy increased to 94.4%, while surgical intervention decreased to 5.7% (p=0.031) (Table 2). No tracheostomies were performed in either period. ICU admission decreased slightly (8.9% vs. 6.8%, p=0.127). Mediastinal complications occurred in six patients (0.6%) during 2006-2014 but were absent in 2015-2023 (p=0.044). Mortality remained extremely low and stable (0.1% vs. 0.1%), with no mediastinitis-related deaths.

Admission labs showed similar WBC counts (13.8 ± 6.0 vs. 13.5 ± 5.9 ×10³/µL, p=0.308). CRP was slightly lower in the later period (45.8 ± 57.6 vs. 54.8 ± 67.0 mg/L, p=0.004), while band form percentages were comparable (2.9 ± 3.9% vs. 2.7 ± 3.0%, p=0.708). Mean hospital stay was similar (6.7 ± 7.1 vs. 6.6 ± 6.0 days, p=0.823).

To evaluate potential selection bias, we compared patients who underwent bacterial culture (n = 243) with those who did not (n = 1,500) (Supplementary Table S2). The cultured group demonstrated significantly higher disease severity, including elevated CRP levels (71.0 vs. 47.8 mg/L), higher surgical rates (30.0% vs. 3.6%), more ICU admissions (19.8% vs. 6.1%), and longer hospital stays (8.4 vs. 6.4 days) (all *p* < 0.001).

To adjust for potential confounders, a multivariable logistic regression analysis was performed (Supplementary Table S3). After adjusting for age, gender, and initial inflammatory markers (WBC and CRP), the later study period (2015-2023) was not an independent predictor for surgical intervention (aOR = 0.75, 95% CI: 0.50-1.12, *p* = 0.168). Instead, elevated initial CRP and WBC levels emerged as the primary independent predictors for both surgical intervention and ICU admission (*p* < 0.001).

To further support the interpretation of period effects, we stratified key outcomes by pediatric stages (< 6 years vs. ≥ 6 years) (Supplementary Table S4). Interestingly, the subgroup analysis revealed that the temporal trend toward decreased surgical intervention was predominantly driven by the older pediatric group (≥ 6 years), where the surgical rate declined significantly from 8.6% to 4.8% (*p* = 0.024). In the younger group (< 6 years), the surgical rate also decreased (8.2% to 6.6%), but without statistical significance (*p* = 0.399). Secondary outcomes (ICU care and hospitalization days) remained relatively stable within both respective age subgroups across the two periods.

### 3.3 Infection Patterns by Microbial Composition

**Figure 2:**
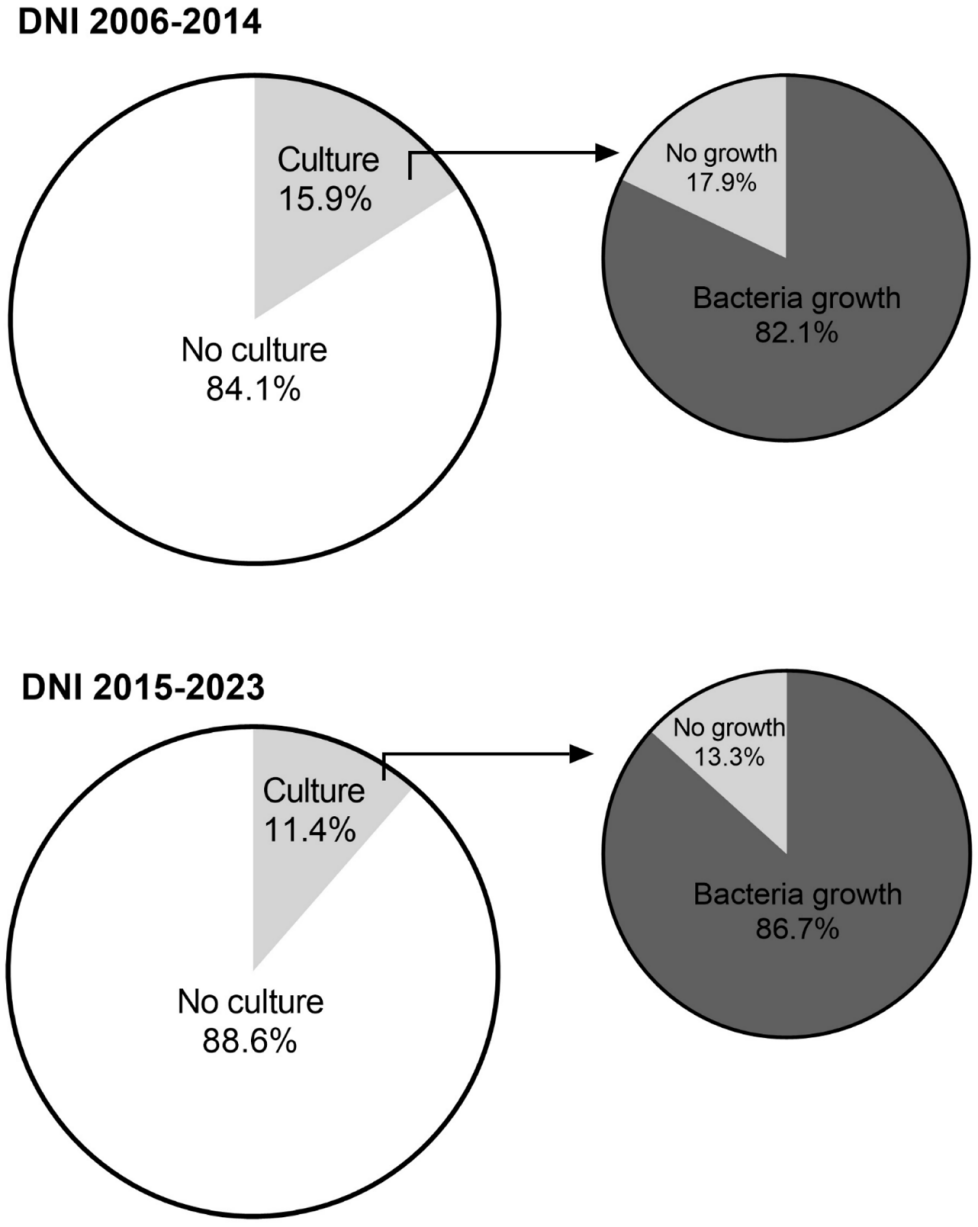
Proportion of Bacterial Cultures and Positive Growth in Pediatric DNI. Pie charts showing the proportion of culture performance and bacterial growth among pediatric DNI patients during two study periods (2006-2014 and 2015-2023).

During 2006-2014, 15.9% of pediatric DNI patients underwent bacterial culture, with 82.1% yielding positive growth (Figure 2). In 2015-2023, culture performance decreased to 11.4%, but positivity increased slightly to 86.7%. In 2006-2014, mono-microbial infections accounted for 51.1% of all cases, dual-microbial for 19.6%, and poly-microbial for 29.3%. In 2015-2023, mono-microbial decreased to 47.9%, dual-microbial increased to 26.8%, and poly-microbial decreased to 25.4%.

Among facultative anaerobic/aerobic isolates, mono-microbial infections declined from 66.4% to 62.1%, while dual-microbial increased from 26.2% to 27.3% and poly-microbial rose from 7.4% to 10.6%. Anaerobic infections showed increased mono-microbial infections (66.7% to74.1%), decreased dual-microbial (31.5% to 26.9%), and negligible poly-microbial cases.

Regarding the sources of these cultures, among the 243 cultured patients, 73 (30.0%) had specimens obtained during formal surgical drainage, while 170 (70.0%) were sampled via needle aspiration or other non-operative deep sampling (Supplementary Table S2).

### 3.4 Genus-Level Analysis of Bacterial Cultures

**Figure 4:**
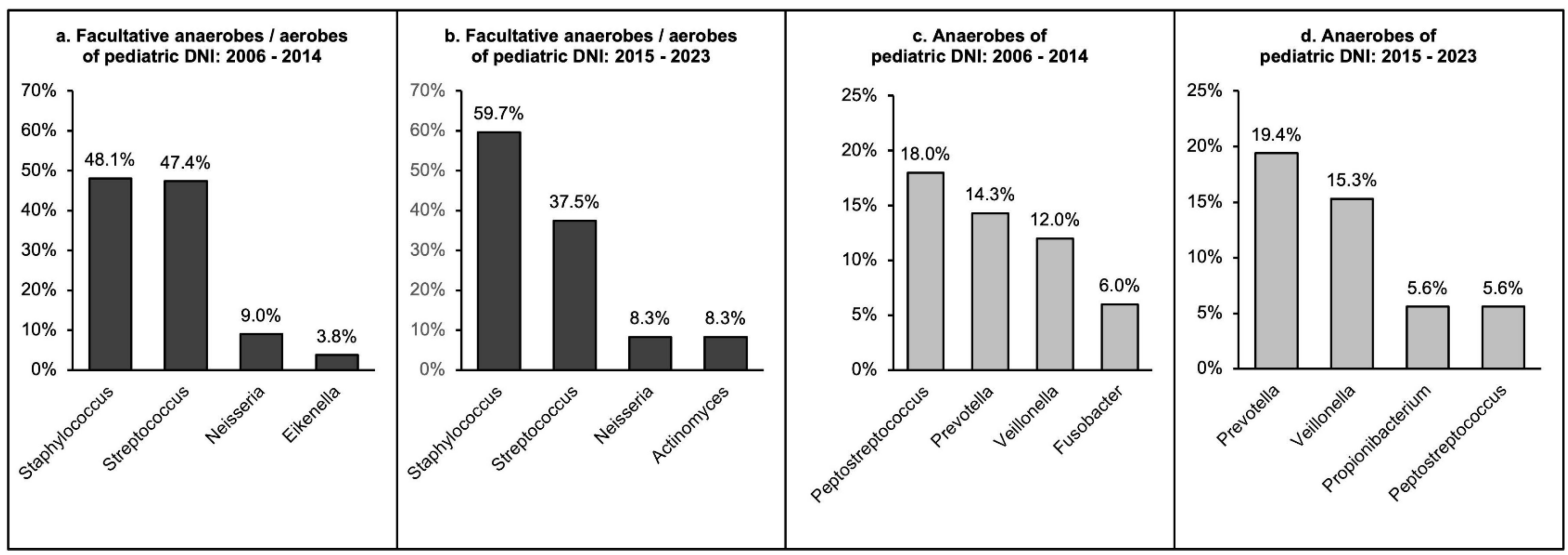
Genus-Level Distribution of Bacterial Isolates in Pediatric DNI. Bar charts showing the genus-level distribution of facultative anaerobic/aerobic (a-b) and anaerobic (c-d) bacterial isolates from pediatric DNI during the two study periods (2006-2014 and 2015-2023). (a, b) Among facultative anaerobic/aerobic bacteria, *Staphylococcus* and *Streptococcus* were the leading genera in both periods. The proportion of *Staphylococcus* increased (48.1% to 59.7%), while *Streptococcus* decreased (47.4% to 37.5%). *Neisseria* remained similar, and *Actinomyces* newly emerged among the top genera in 2015-2023. (c, d) Among anaerobes, *Peptostreptococcus* predominated in 2006-2014 (18.0%) but decreased markedly by 2015-2023 (5.6%), while *Veillonella* rose (12.0% to 15.3%) and *Prevotella* became the most common genus (19.4%) in the later period. *Propionibacterium* newly appeared among the top four anaerobic genera.

In 2006-2014, *Staphylococcus* (48.1%) and *Streptococcus* (47.4%) were the most frequent facultative anaerobes/aerobes, followed by *Neisseria* (9.0%) and *Eikenella* (3.8%). In 2015-2023, *Staphylococcus* increased to 59.7%, *Streptococcus* decreased to 37.5%, *Neisseria* remained similar (8.3%), and *Actinomyces* (8.3%) replaced *Eikenella* (Figure 4).

For anaerobes, *Peptostreptococcus* (18.0%), *Prevotella* (14.3%), and *Veillonella* (12.0%) predominated in 2006-2014, with *Fusobacterium* at 6.0%. In 2015-2023, *Prevotella* became the leading anaerobic genus (19.4%), followed by *Veillonella* (15.3%), while *Propionibacterium* appeared (5.6%) and *Peptostreptococcus* decreased to 5.6%.

### 3.5 Species-Level Analysis of Bacterial Cultures

**Figure 5:**
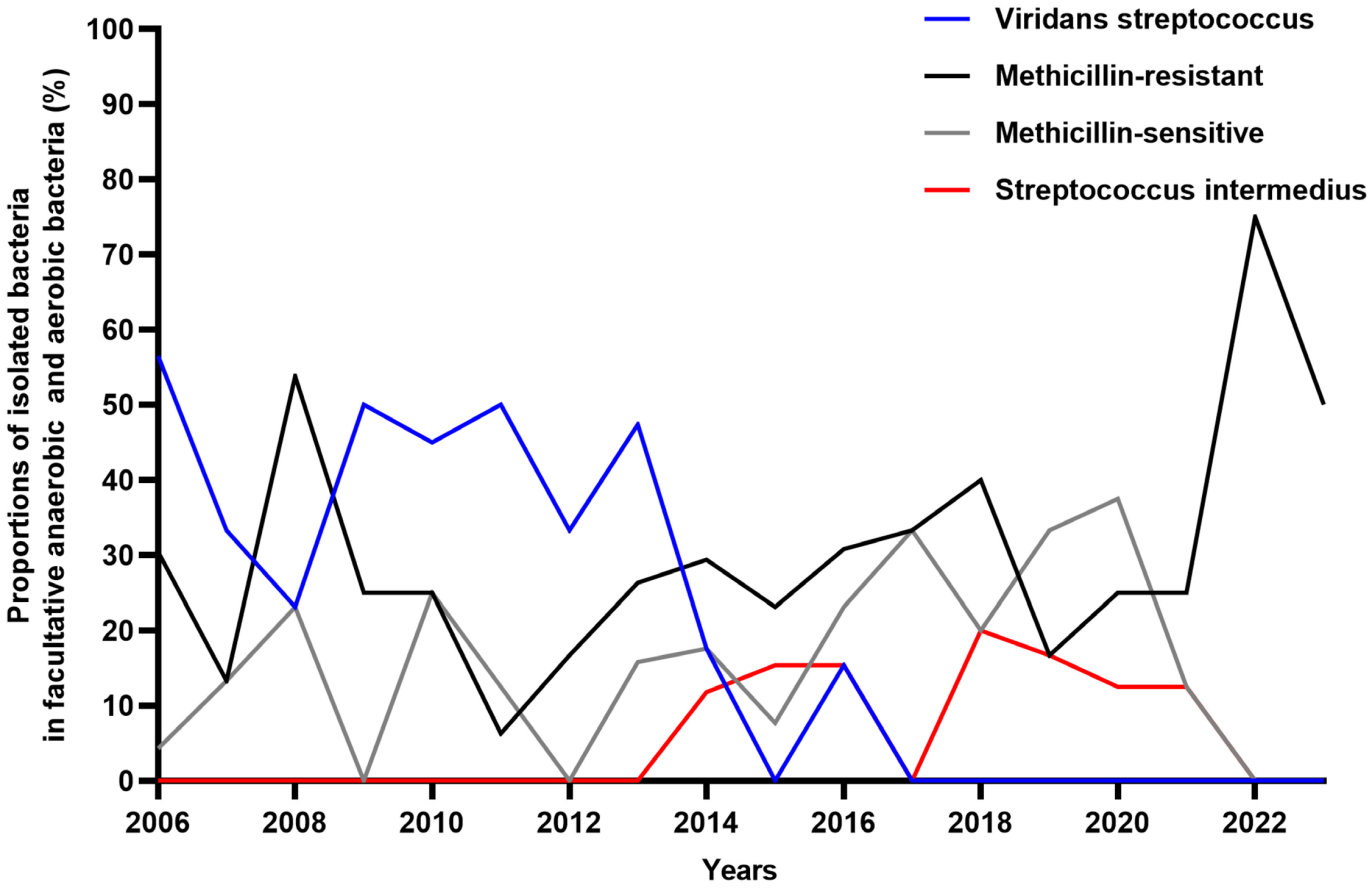
** Temporal Trends of Major Facultative Anaerobic and Aerobic Bacterial Species in Pediatric DNI, 2006-2023.** Longitudinal trends showing proportions of the top facultative anaerobic and aerobic bacterial species isolated from pediatric DNI cases over 18 years. *Viridans streptococcus* (blue line) predominated in the early period (2006-2014) but declined sharply after 2014. *Methicillin-resistant Staphylococcus aureus* (MRSA; black line) increased markedly after 2014 and became the most frequent isolate in 2015-2023. *Methicillin-sensitive S. aureus* (MSSA; gray line) remained relatively stable with moderate fluctuations, while *Streptococcus intermedius* (red line) appeared later and persisted at low levels. *Cautionary Note: Annual fluctuations reflect small sample sizes; focus should remain on the overarching long-term shifts across the two study periods rather than individual year peaks.*

**Figure 6:**
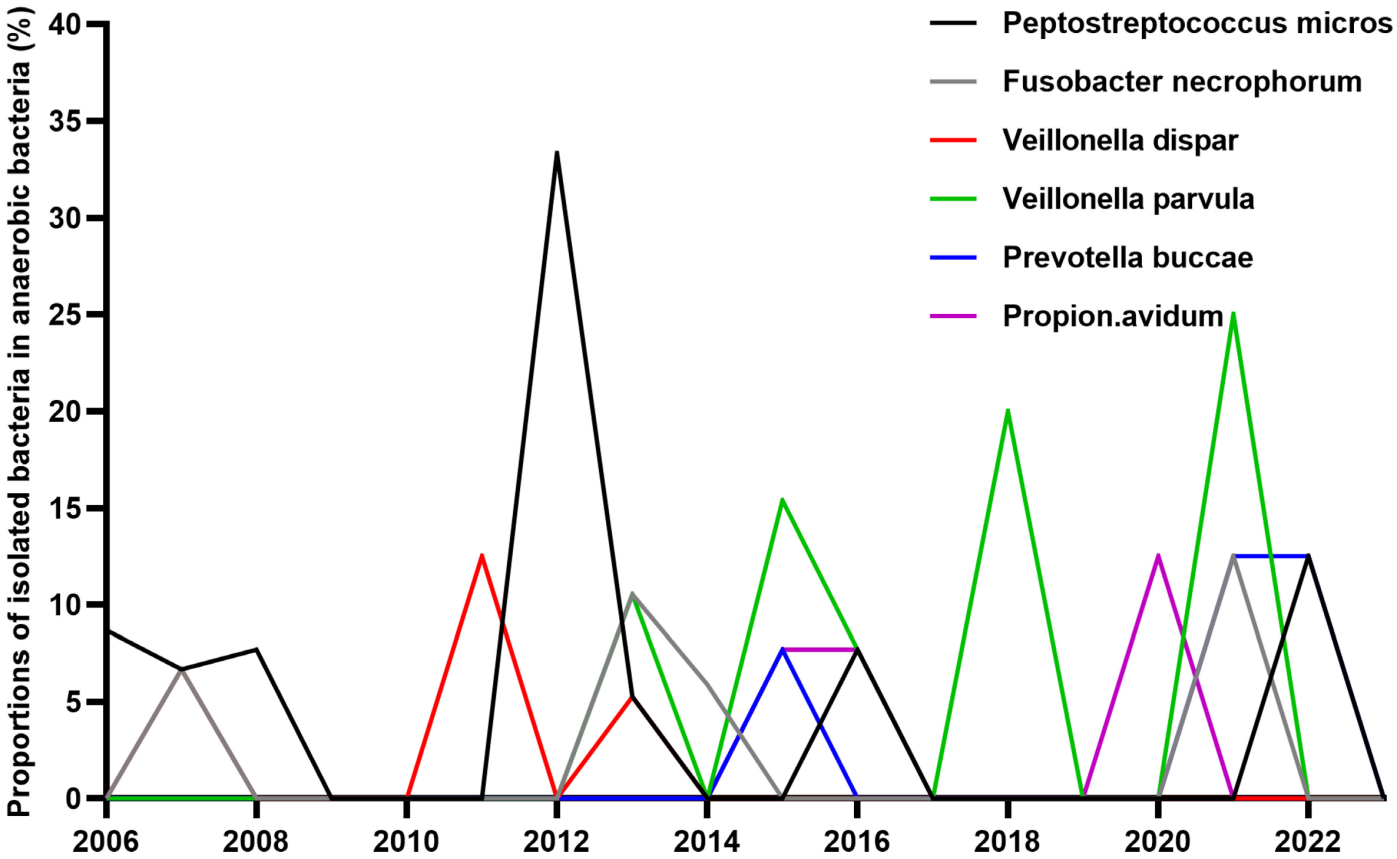
** Temporal Trends of Major Anaerobic Bacterial Species in Pediatric DNI, 2006-2023.** Line graph demonstrating the longitudinal distribution of predominant anaerobic bacteria isolated from pediatric DNI cases across the 18-year study period. *Peptostreptococcus micros* (black) predominated during the early years but gradually declined after 2015. *Veillonella parvula* (green) increased steadily during 2015-2023, peaking in 2021 and becoming the most frequent anaerobic isolate in the later period. Other species—including *Fusobacterium necrophorum* (gray), *Prevotella buccae* (blue), *Veillonella dispar* (red), and *Propionibacterium avidum* (purple)—appeared sporadically over time with relatively low prevalence. *Cautionary Note: Annual fluctuations reflect small sample sizes; focus should remain on the overarching long-term shifts across the two study periods rather than individual year peaks.*

We identified the top three species of facultative anaerobic/aerobic and anaerobic bacteria in each time period and examined their longitudinal changes (Figures 5 and 6). Because antibiotic selection differs substantially between methicillin-sensitive *Staphylococcus aureus* (MSSA) and methicillin-resistant *S. aureus* (MRSA), we analyzed them separately. For facultative anaerobic/aerobic bacteria, the top three species during 2006-2014 were *viridans streptococcus* (40.6%), MRSA (25.6%), and MSSA (14.3%). In 2015-2023, MRSA became the leading isolate (33.8%), followed by MSSA (18.3%) and *Streptococcus intermedius* (11.3%). Over the entire study period, *viridans streptococcus* showed a steady decline, MRSA increased markedly after 2014. While peaks were observed in 2021 for both MRSA and *V. parvula*, these should be interpreted as part of the overarching longitudinal shifts during 2015-2023 rather than isolated annual events.

For anaerobic bacteria, the top three species in 2006-2014 were *Peptostreptococcus micros* (5.3%), *Fusobacterium necrophorum* (3.0%), and *Veillonella dispar* (3.0%). In 2015-2023, *Veillonella parvula* became the most common anaerobic species (9.9%), followed by *Prevotella buccae* (4.2%) and *Propionibacterium avidum* (4.2%). *P. micros* predominated in the earlier period but decreased thereafter, whereas *V. parvula* increased steadily during 2015-2023 and peaked in 2021. Other species—including *F. necrophorum*, *P. buccae*, *V. dispar*, and *P. avidum*—appeared sporadically throughout the study.

In a sensitivity analysis excluding the COVID-19 pandemic years (2020-2022), the longitudinal shifts toward MRSA and *Veillonella parvula* predominance remained evident, confirming these as sustained long-term trends rather than transient pandemic artifacts (Supplementary Table S1).

## 4. Discussion

In this large, multicenter study using the CGRD, we analyzed 1,743 pediatric DNI cases over an 18-year period and, for the first time, provide a comprehensive picture of temporal changes in demographics, treatment patterns, disease severity, and pathogen distribution in Taiwanese children. Our findings reveal clear shifts in both clinical management and microbial composition, offering updated evidence to guide empiric antibiotic therapy and optimize the care of pediatric DNI.

We observed a clear temporal shift in the management of pediatric DNI between 2006-2014 and 2015-2023, with the proportion of children treated with antibiotics alone increasing from 91.5% to 94.4%, whereas the rate of surgical intervention decreased from 8.5% to 5.7%. Importantly, this high rate of non-operative resolution should not be interpreted causally as conservative management driving better outcomes, given the inherent confounding by indication (i.e., milder cases are preferentially selected for antibiotic-only therapy). Rather, this trend reflects that contemporary diagnostic and antimicrobial strategies allow a larger proportion of pediatric DNI cases to be safely managed without operative drainage, without compromising patient safety. Previous pediatric studies have reported similar findings. Craig and Schunk (2003) observed that 58% of 64 children with retropharyngeal abscesses were successfully treated with intravenous antibiotics alone, without any treatment failures 11, while Metin *et al*. (2014) found that 64% of pediatric DNI cases in Turkey resolved with medical therapy alone 12. Extending beyond these single-center experiences, a nationwide U.S. analysis by Novis *et al*. (2014) demonstrated that from 2000 to 2009, the incidence of patients undergoing surgical drainage declined from 48% to 38% (p = 0.04) 13. Compared with these earlier reports, our 18-year multicenter study not only corroborates this long-term global shift but also demonstrates an even higher success rate for antibiotic-only therapy (>90%) and extremely low mortality (0.1%) without mediastinitis-related deaths. Despite the declining rate of surgical intervention, ICU admission rates and laboratory markers of systemic inflammation (WBC counts and band forms) remained relatively stable, and mediastinal complications became less frequent in the later period (0.6 to 0%). These findings confirm that modern management strategies—characterized by timely diagnosis, broad-spectrum empiric antibiotics, and selective surgical drainage—achieve excellent outcomes in children with DNI while minimizing surgical morbidity 14.

Our findings also reveal distinct temporal changes in the infection patterns and microbial composition of pediatric DNI. Although the overall culture positivity rate remained relatively stable between the two study periods, the proportion of patients undergoing bacterial culture decreased, which may reflect changes in clinical practice such as earlier empiric therapy or a lower threshold for initiating antibiotics prior to specimen collection. In parallel, the composition of isolates shifted: mono-microbial infections remained predominant, but dual-microbial infections among facultative anaerobes/aerobes increased in the later period, while anaerobic infections tended toward a higher proportion of mono-microbial isolates. These changes likely mirror evolving epidemiology in the community and improvements in diagnostic technology. Specifically, the observed decline in *Streptococcus* species and the concurrent rise in *Staphylococcus aureus* (including MRSA) are likely linked to the impact of national immunization strategies. In January 2015, Taiwan integrated the 13-valent pneumococcal conjugate vaccine (PCV13) into its routine pediatric National Immunization Program. The widespread uptake of PCV13 not only significantly reduced the nasopharyngeal carriage of vaccine-type streptococci but also likely triggered an “ecological niche replacement.” Given the well-documented inverse relationship between pneumococcal and *S. aureus* colonization 15,16, the vaccine-induced void in the upper aerodigestive tract provides a strong biological rationale for the subsequent emergence of *Staphylococcus* predominance during the 2015-2023 period.

The distinct peaks for MRSA and *V. parvula* around 2021 likely reflect the impact of the COVID-19 pandemic. During this period, fear of hospital exposure often led to delayed presentations, resulting in more severe or refractory infections that are inherently more likely to harbor resistant (MRSA) or complex endogenous flora (*Veillonella*) 17,18. However, our sensitivity analysis (Supplementary Table S1), which excluded the pandemic years (2020-2023), confirmed that the broader ecological transition began well before the pandemic, representing a genuine long-term trend rather than a transient COVID-19 artifact.

Our study identified a clear longitudinal shift in the bacterial composition of pediatric DNI over the past 18 years, characterized by a growing predominance of *Staphylococcus* and a corresponding decline of *Streptococcus* among facultative anaerobic and aerobic genera. In contrast, the anaerobic community showed a distinct redistribution pattern, with *Veillonella* progressively increasing while *Peptostreptococcus* declined, indicating that *Veillonella* has become the predominant anaerobic genus in recent years. Previous pediatric DNI studies consistently identified *Staphylococcus* and *Streptococcus* as the major genera, but their relative proportions varied across time and geography. Coticchia *et al*. reported *Staphylococcus aureus* dominance in infants and *Streptococcus pyogenes* in older children, reflecting an age-dependent distribution 19. Shimizu *et al*. later found *Staphylococcus* species in 60% and *Streptococcus* in 27% of pediatric cases in Japan 4, while Kharel *et al*. documented *Staphylococcus* as the most frequent isolate in a recent Nepali cohort 3. These findings collectively align with our observation of *Staphylococcus* predominance but lacked long-term trend data. By spanning nearly two decades, our analysis captured both the earlier coexistence of *Staphylococcus* and *Streptococcus* and their subsequent divergence toward *Staphylococcus* dominance—indicating an evolving microbial landscape possibly driven by antibiotic selection pressure, vaccination, or changes in community carriage patterns.

At the species level, our findings further delineate the dynamic microbial evolution underlying pediatric DNI. During 2006-2014, *viridans streptococci* represented the dominant facultative anaerobic/aerobic species, whereas MRSA became increasingly prevalent after 2014, surpassing *viridans streptococci* to become the leading isolate in 2015-2023. Earlier investigations seldom addressed species-level variations over time; most reports were limited by small sample sizes and narrow temporal scopes. Coticchia *et al*. (2004) observed that *S. aureus* predominated in infants younger than 1 year, while *group A streptococcus* was more common in older children, indicating an age-dependent microbial distribution 19. Our long-term, multicenter dataset extends these observations by capturing a continuous shift from *viridans streptococci* toward MRSA dominance, reflecting evolving antimicrobial exposure and community transmission dynamics in the pediatric population. For anaerobes, *Peptostreptococcus micros* was the leading isolate in 2006-2014 but gradually declined thereafter, while *Veillonella parvula* increased steadily and became the most frequent anaerobic species after 2020. Previous pediatric reports rarely differentiated specific anaerobic taxa, often referring to “mixed anaerobic infections” collectively without species identification (e.g., Coticchia *et al*., 2004) 19. Our data thus provide novel evidence of an ecological transition from gram-positive anaerobes (*Peptostreptococcus*) toward gram-negative commensal-derived organisms (*Veillonella*). Importantly, the epidemiological shift toward *Veillonella* is linked to advancements in laboratory diagnostics. Our hospital network fully adopted Matrix-Assisted Laser Desorption/Ionization Time-of-Flight Mass Spectrometry (MALDI-TOF MS) between 2013 and 2014, which drastically improved the sensitivity of anaerobic species identification compared to older biochemical systems. Therefore, the emergence of *V. parvula* in the later period likely reflects both a true ecological evolution and the diagnostic 'unmasking' of its prevalence that was previously underreported.

Although *V. parvula* emerged as the leading anaerobe in the later period, it is important to note that it is primarily an oral commensal with low standalone virulence. Its clinical significance in pediatric DNI likely stems from its role as a synergistic co-pathogen within polymicrobial infections. *Veillonella* species are known to facilitate multi-species biofilm formation and provide metabolic support (e.g., via lactic acid consumption) that enhances the survival and pathogenic potential of more aggressive co-pathogens, such as *Staphylococcus* or *Streptococcus* 20. Therefore, the prominent isolation of *V. parvula* frequently signals a complex, synergistic abscess microenvironment rather than a highly virulent monomicrobial infection.

Given the high success rate of standard medical therapy for mild cases, our findings highlight that empiric antibiotic selection should carefully consider coverage of MRSA and emerging anaerobes such as *Veillonella parvula* specifically for severe, refractory, or surgically managed pediatric DNIs where typical first-line regimens have failed or clinical presentation is critical.

The unexpectedly high proportion of monomicrobial infections, particularly among anaerobes, likely reflects clinical and laboratory practices rather than true biological monoculture. First, most patients received empiric antibiotics prior to culture, which can selectively suppress susceptible strains, allowing only dominant or resistant pathogens to be isolated. Second, prior to the standardization of MALDI-TOF MS, institutional reporting rules for complex cultures often used terms like 'mixed anaerobes' when individual species separation was unfeasible. Consequently, the true prevalence of polymicrobial anaerobic involvement is likely underestimated in our species-level data.

The lower-case volume in the later period (726 vs. 1,017) likely mirrors Taiwan's declining birth rate and shrinking pediatric population rather than a true decline in DNI incidence. Accordingly, our analysis focused on the proportional shifts in treatment and microbiology within the DNI cohort over time.

Several limitations of this study should be acknowledged. First, although the CGRD is a large, multi-institutional database, relying on ICD codes introduces the risk of coding inaccuracies. To mitigate this, our operational definition strictly required inpatient hospitalization corroborated by concurrent clinical actions (e.g., intravenous antibiotics, operative procedures, or cultures). Nevertheless, residual misclassification—such as inadvertently coding severe superficial soft tissue infections as DNI—could potentially underestimate overall disease severity and skew the microbiological spectrum toward typical skin flora. Furthermore, the under-coding of secondary diagnoses may underestimate true comorbidity prevalence. Second, bacterial culture was performed in only a minority of patients (11.4%-15.9%). Our comparative analysis (Supplementary Table S2) confirms that cultured patients had significantly higher disease severity and surgical rates. Consequently, our microbiological findings predominantly represent the etiology of severe, refractory, or surgically managed DNI cases rather than mild ones. Third, due to the nature of this multi-institutional database spanning 18 years, comprehensive antibiotic susceptibility profiles (e.g., detailed antibiograms for all species) were not uniformly standardized or available for extraction. However, to address the most critical clinical concern regarding antimicrobial resistance, we explicitly stratified *Staphylococcus aureus* isolates into MRSA and MSSA strains. Fourth, the relatively small annual numbers of some anaerobic isolates produced jagged year-to-year trends. Therefore, single-year spikes (e.g., during 2021) should not be over-interpreted; our analysis robustly highlights the broader, long-term ecological shifts across the two 9-year periods. Finally, most specimens (70.0%) were collected via needle aspiration rather than surgical drainage (30.0%). Since needle aspiration is less effective at preserving sensitive anaerobic bacteria, our cultures likely missed some of these pathogens. Therefore, the actual prevalence of anaerobes like *Veillonella* is probably higher than our data indicates.

### What This Study Means

- This 18-year multicenter cohort provides contemporary evidence describing stable clinical outcomes alongside gradual shifts in microbial composition.
- The increasing detection of MRSA and anaerobes such as *Veillonella parvula* suggests evolving bacterial patterns in recent years.
- These findings enhance current understanding of pediatric DNI epidemiology and support more evidence-based decisions in empiric antibiotic management.

## 5. Conclusions

This 18-year multicenter study demonstrates clear temporal shifts in the microbial spectrum and treatment patterns of pediatric DNI in Taiwan. MRSA has emerged as the predominant facultative anaerobic/aerobic pathogen, while *Veillonella parvula* has become the leading anaerobic species. Despite these microbiological changes, disease severity has remained stable and mortality extremely low under contemporary management strategies. These updated data provide essential evidence to guide empiric antibiotic selection, particularly emphasizing the need to consider MRSA and *Veillonella* coverage in complicated or severe cases that necessitate surgical intervention or fail to respond to initial medical management.

## Supplementary Material

Supplementary tables.

## Figures and Tables

**Figure 3:**
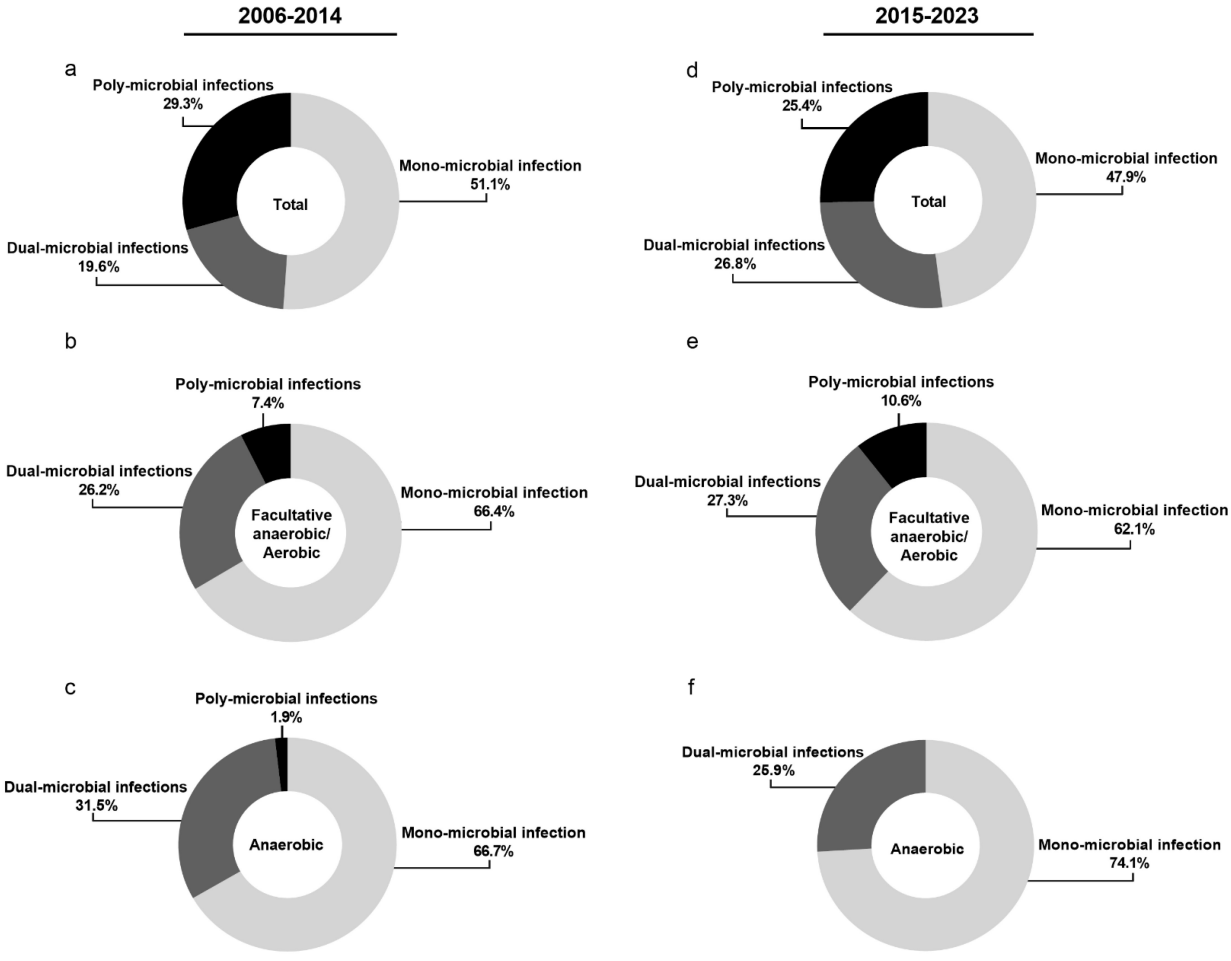
** Infection Patterns by Microbial Composition in Pediatric DNI.** Distribution of mono-, dual-, and poly-microbial infections among pediatric DNI patients during the two study periods (2006-2014 and 2015-2023). (a-c) represent total, facultative anaerobic/aerobic, and anaerobic infections, respectively, for 2006-2014; (d-f) represent the same categories for 2015-2023.

**Table 1:** Baseline demographics and comorbidities of pediatric DNI patients stratified by study period (2006-2014 [n = 1,017] vs. 2015-2023 [n = 726]).

	2006-2014		2015-2023		
Variables	n	%	n	%	p-value*
Total	1017		726		
Age (mean ± SD)†	7.1 ± 4.8		6.7 ± 4.4		0.082
Gender					0.807
Male	573	56.3	404	55.7	
Female	444	43.7	322	44.4	
Covariates					
CKD	0	0.0	1	0.1	0.417
DM	1	0.1	6	0.8	0.023
LC	2	0.2	3	0.4	0.655
RA	1	0.1	1	0.1	1.000
SS	0	0.0	2	0.3	0.173
SLE	3	0.3	2	0.3	1.000

Continuous data: mean ± SD; categorical data: n (%). *Fisher's exact test; †Student's t-test. Abbreviations: DNI, deep neck infection; CKD, chronic kidney disease; DM, diabetes mellitus; LC, liver cirrhosis; RA, rheumatoid arthritis; SS, Sjogren syndrome; SLE, systemic lupus erythematosus; SD, standard deviation.

**Table 2:** Treatment, disease severity, and clinical outcomes of pediatric DNI patients stratified by study period (2006-2014 vs. 2015-2023).

	2006-2014		2015-2023		
Variables	n	%	n	%	*p*-value^*^
Total	1017		726		
Therapy					0.031
Antibiotic ± Aspiration	931	91.5	685	94.4	
Surgery	86	8.5	41	5.7	
Tracheostomy	0	0.0	0	0.0	-
ICU^a^ care	90	8.9	49	6.8	0.127
Mediastinitis	6	0.6	0	0.0	0.044
Prognosis					
Mediastinitis-Mortality	0	0.0	0	0.0	-
Mortality without tracheostomy	1	0.1	1	0.1	1.000
Mortality	1	0.1	1	0.1	1.000
	Mean ± SD		Mean ± SD		*p*-value^†^
Hospitalization (days)	6.6 ± 6.0		6.7 ± 7.1		0.823
Lab variables					
WBC^b^, 10^3^/uL	13.8 ± 6.0		13.5 ± 5.9		0.308
CRP^c^, mg/l	54.8 ± 67.0		45.8 ± 57.6		0.004
Band form, %	2.7 ± 3.0		2.9 ± 3.9		0.708

'Antibiotic ± Aspiration': intravenous antibiotics ± needle aspiration without surgery; 'Surgery': formal incision and drainage. Laboratory values (WBC, CRP, band form) represent admission measurements. Continuous data: mean ± SD; categorical data: n (%). *Fisher's exact test; †Student's t-test. Abbreviations: ICU, intensive care unit; WBC, white blood cell count; CRP, C-reactive protein; SD, standard deviation.
