# Supplementary material for: Long-Term Trends in Microbiology and Outcomes of Pediatric Deep Neck Infections: An 18-Year Study
Source: Int J Med Sci. 2026 Jul 22;23(8):2728–38. doi: 10.7150/ijms.131187 (PMC13411609; doi:10.7150/ijms.131187)
Supplement: Supplementary file 1 — Supplementary tables. [file ijmsv23p2728s1.pdf]

Pathogen Category	Bacterial Species	Later Period (2015–2023) (%)	Sensitivity Analysis (2015–2019) (%)
**Facultative anaerobes / Aerobes**	MRSA	33.8%	27.9%
	MSSA	18.3%	20.9%
	Streptococcus intermedius	11.3%	14.0%
**Anaerobes**	*Veillonella parvula*	9.9%	9.3%
	Prevotella buccae	4.2%	—
	Prevotella bivia	—	4.7%
	Propionibacterium avidum	4.2%	4.7%

Supplementary Table S1. Sensitivity analysis of the top three bacterial species in culture-positive pediatric DNI patients, comparing the full later period (2015–2023) with a pre-pandemic interval (2015–2019), excluding COVID-19 pandemic years. Values: proportion (%) of each species among all isolates; a dash (–) indicates the species was not among the top three. Abbreviations: MRSA, methicillin-resistant Staphylococcus aureus; MSSA, methicillin-sensitive Staphylococcus aureus.

	Cultured		Non-cultured		
	n	%	n	%	*p*-value[*]
Total	243		1500		
Gender					0.118
Male	125	51.4	852	56.8	
Female	118	48.6	648	43.2	
Age (M ± SD)[†]	7.0 ± 5.9		7.0 ± 4.4		0.951
Therapy					<0.001
Antibiotic ± Aspiration	170	70.0	1446	96.4	
Surgery	73	30.0	54	3.6	
Hospitalization (Days)	8.4 ± 5.8		6.4 ± 6.6		<0.001
ICU care	48	19.8	91	6.1	<.0001
Laboratory					
WBC, 10^3/μL (M ± SD)[†]	16.7 ± 6.9		13.2 ± 5.6		<0.001
CRP, mg/L (M ± SD)[†]	71.0 ± 70.0		47.8 ± 61.7		<0.001

Supplementary Table S2. Comparison of clinical characteristics between culture-positive (n = 243) and culture-negative (n = 1,500) pediatric DNI patients (2006–2023). Among cultured patients, 73 (30.0%) had specimens from surgical drainage and 170 (70.0%) from non-operative sampling.

Continuous data: mean ± SD; categorical data: n (%). *Chi-squared test; †Student's t-test. Abbreviations: ICU, intensive care unit; WBC, white blood cell count; CRP, C-reactive protein; SD, standard deviation.

Variables	Surgical intervention			ICU admission		
	**aOR**	**95% CI**	**p-value**	**aOR**	**95% CI**	**p-value**
**Time period**						
2006–2014	1.00	(Reference)		1.00	(Reference)	
2015–2023	0.75	0.50–1.12	0.168	0.99	0.65–1.52	0.976
**Age (years)**	1.00	0.95–1.04	0.921	1.05	1.01–1.11	0.032
**Gender (Male)**	1.01	0.68–1.49	0.970	0.91	0.60–1.37	0.664
**WBC (10^3/µL)**	1.08	1.04–1.11	< 0.001	1.11	1.08–1.15	< 0.001*
**CRP (mg/L)**	1.08	1.05–1.11	< 0.001	1.10	1.06–1.12	0.001

Supplementary Table S3. Multivariable logistic regression for independent predictors of surgical intervention and ICU admission in all pediatric DNI patients (n = 1,743; 2006–2023). Both models adjusted for age, sex, study period, WBC, and CRP. Results expressed as adjusted odds ratios (aOR) with 95% CI. Abbreviations: aOR, adjusted odds ratio; CI, confidence interval; WBC, white blood cell count; CRP, C-reactive protein; ICU, intensive care unit.

Variables	2006–2014		2015–2023		p-value
	n	%	n	%	
**Preschool age (< 6 years)**	N = 449		N = 332		
Surgery*	37	8.2	22	6.6	0.399
ICU care*	52	11.6	30	9.0	0.251
Hospitalization (days)†	6.6 ± 6.0		7.3 ± 9.1		0.218
**School age (≥ 6 years)**	N = 568		N = 394		
Surgery*	49	8.6	19	4.8	0.024
ICU care*	38	6.7	19	4.8	0.228
Hospitalization (days)†	6.6 ± 6.0		6.2 ± 4.8		0.184

Supplementary Table S4. Subgroup analysis of surgical intervention, ICU admission, and hospitalization duration by pediatric age group (< 6 years vs. ≥6 years) and study period (2006－2014 vs. 2015－2023). Categorical data: n (%); *Pearson’ s chi-square test. Continuous data: mean ± SD; †Student’s t-test. Abbreviations: ICU, intensive care unit; SD, standard deviation.
